# Multiple E-Boxes in the Distal Promoter of the Rat Pyruvate Carboxylase Gene Function as a Glucose-Responsive Element

**DOI:** 10.1371/journal.pone.0102730

**Published:** 2014-07-23

**Authors:** Apilak Wutthisathapornchai, Tuangtong Vongpipatana, Sureeporn Muangsawat, Thirajit Boonsaen, Michael J. MacDonald, Sarawut Jitrapakdee

**Affiliations:** 1 Department of Biochemistry, Faculty of Science, Mahidol University, Bangkok, Thailand; 2 UW Childrens Diabetes Center, University of Wisconsin School of Medicine and Public Health, Madison, WI, United States of America; North Carolina State University, United States of America

## Abstract

Pyruvate carboxylase (PC) is an anaplerotic enzyme that regulates glucose-induced insulin secretion in pancreatic islets. Dysregulation of its expression is associated with type 2 diabetes. Herein we describe the molecular mechanism underlying the glucose-mediated transcriptional regulation of the PC gene. Incubation of the rat insulin cell line INS-1 832/13 with glucose resulted in a 2-fold increase in PC mRNA expression. Transient transfections of the rat PC promoter-luciferase reporter construct in the above cell line combined with mutational analysis indicated that the rat PC gene promoter contains the glucose-responsive element (GRE), comprising three canonical E-boxes (E1, E3 and E4) and one E-box-like element (E2) clustering between nucleotides –546 and –399, upstream of the transcription start site. Mutation of any of these E-boxes resulted in a marked reduction of glucose-mediated transcriptional induction of the reporter gene. Electrophoretic mobility shift assays revealed that the upstream stimulatory factors 1 and 2 (USF1 and USF2) bind to E1, the Specificity Protein-1 (Sp1) binds to E2, USF2 and the carbohydrate responsive element binding protein (ChREBP) binds to E4, while unknown factors binds to E3. High glucose promotes the recruitment of Sp1 to E2 and, USF2 and ChREBP to E4. Silencing the expression of Sp1, USF2 and ChREBP by their respective siRNAs in INS-1 832/13 cells blunted glucose-induced expression of endogenous PC. We conclude that the glucose-mediated transcriptional activation of the rat PC gene is regulated by at least these three transcription factors.

## Introduction

Glucose homeostasis is tightly regulated by glucagon and insulin which counteract each other to maintain the concentration of plasma glucose within a narrow range. Glucagon secreted during prolonged starvation raises the blood glucose level by stimulating glycogen breakdown and gluconeogenesis [1] while insulin secreted during fed conditions [glucose-induced insulin secretion (GSIS)] lowers blood glucose levels by stimulating glucose uptake, glycolysis and glycogen synthesis in liver and skeletal muscle [2]. This process occurs efficiently in the pancreatic β-cell due to the presence of GLUT2 and glucokinase [3], which together acts as a sensor allowing high concentrations of glucose to enter to the cell for aerobic glycolysis and oxidative phosphorylation [4]. These biochemical pathways raise the concentrations of cellular ATP which drive insulin secretion known as K_ATP_-dependent GSIS [5]. Although pyruvate formed after glycolysis appears to enter beta cell mitochondria through pyruvate decarboxylation catalyzed by pyruvate dehydrogenase complex (PDH) and pyruvate carboxylation by pyruvate carboxylase (PC) with similar proportions, flux toward the latter reaction is tightly associated with the glucose concentration the cells is exposed to and thus correlates with the magnitude of insulin secretion [6], [7]. In further support of this observation, PC protein levels in rat pancreatic islets were found to be rapidly induced by exogenous glucose [8]. Subsequent studies have clearly revealed that indeed pyruvate carboxylation by PC constitutes an important component of the “pyruvate cycling” which provides NADPH, one of coupling factors required for GSIS in pancreatic β-cells [9]–[11]. Suppression of PC expression in rat insulinoma cells impairs anaplerosis, concomitant with the lowered GSIS [12]–[13] while overexpression of PC enhances GSIS [13], indicating the critical role of PC in supporting insulin secretion. Several studies performed in models of diabetic rats and human subjects with type 2 diabetes clearly show that down-regulation of PC expression in pancreatic islets is associated with type 2 diabetic phenotypes further supporting the role of PC in GSIS [14]–[16].

In the rat, mouse, and human, PC expression is regulated by two alternative promoters, the proximal promoter is active in liver and adipose tissue where PC is involved in gluconeogenesis and lipogenesis, respectively while the distal promoter is active in β-cells where PC is required to support GSIS [17]–[20]. Characterization of the distal promoter of rat PC gene revealed that a member of the forkhead box protein 3β (FoxA2) regulates PC expression in a β-cell specific manner [21]. Although previous work has shown that PC expression is inducible by exogenous glucose, it remains largely unknown how glucose increases PC expression [8], [17].

Specificity protein 1 (Sp1) is a zinc-finger and ubiquitously expressed transcription factor which regulates expression of a variety of genes. Sp1 can regulate transcription of several metabolic genes in response to physiological changes including alterations in glucose levels. High glucose stimulates activity of protein phosphatase 1 which in turn dephosphorylates Sp1, enhancing its DNA binding activity [22]. The upstream stimulatory factors (USFs), comprising USF1 and USF2, and the carbohydrate response element binding protein (ChREBP) are the basic helix-loop-helix leucine zipper transcription factor. While USFs as a homo- or heterodimer recognize the canonical E-box (CANNTG), ChREBP is heterodimerized with Mlx, and bind to two adjacent E-boxes, termed the carbohydrate responsive element (ChoRE) in their target genes. Elevated glucose concentrations increase the abundance of ChREBP mRNA [23] and the activity via xylulose-5-phosphate, an intermediate of the pentose phosphate pathway by stimulating the activity of protein phosphatase 1A which in turn dephosphorylates ChREBP, enhancing its entry into nucleus [24]. Furthermore, a conformational change within the glucose-sensing module of ChREBP induced by high glucose also contributes to its ability to transactivate its target genes [25].

Although Pedersen *et al*. [26] have previously reported that ChREBP regulates glucose-induced PC expression via the −408/−392 ChoRE, locating in the distal promoter of rat PC gene, a weak binding of ChREBP to this ChoRE has been observed. Moreover most of this study was performed using a heterologous promoter-reporter and the functional importance of this transcription factor with respect to transcriptional regulation of endogenous PC expression was not demonstrated. Here we identify the glucose-responsive element (GRE) within the 1 kb enhancer region of rat PC gene that mediates transcriptional induction of PC by glucose. Our results reveal that the ChREBP, USF2 and Sp1 transcription factors act in concert via binding to the tandem E-boxes located within the first 600 nucleotides upstream of the transcription start site. We also confirmed the functional importance of these three transcription factors with respect to the regulation of glucose-induced expression of the endogenous PC gene with gene knockdown experiments.

## Materials and Methods

### Plasmids construction

The construction of the 1.2 kb 5′-flanking region representing the distal promoter of rat PC gene-luciferase reporter construct (pGL-P2) and its 5′-truncated mutant constructs were described previously [27]. The pGL-P2 reporter construct with various E-boxes mutated, namely, MuE1, MuE2, MuE3, MuE4 were constructed using pGL-P2 as a template while the double mutant MuE2&E4 was constructed using MuE4 as the template. The pGL-P2ΔKpnI mutants containing 25 bp or 23 bp internal deletions (Δ1, Δ2, Δ3, Δ4, Δ5 and Δ6) were constructed using pGL-P2ΔKpnI as template. The mutagenic reaction was performed using Quik-change XL site-directed mutagenesis kit (Stratagene) following the manufacturer’s instructions. The mutagenic primers used to generate the above constructs are shown in Table1. The nucleotide sequences of the mutagenic clones were verified by automated DNA sequencing (Macrogen Inc, USA).

The plasmids encoding USF1 and USF2 were cloned from cDNA prepared from INS-1 832/13 cells by RT-PCR using the primers (USF1CDS F/R and USF2CDS F/R) designed from published cDNA sequences [28]. The USF1 and USF2 cDNAs were cloned into the *Hind*III and *Kpn*I sites of pcDNA3 expression vector (Invitrogen). Plasmids encoding Sp1 and Sp3 were constructed as described previously [29].

### Cell culture and transient transfection

INS-1 and INS-1 832/13 cell lines [30] were kindly provided by C.B. Newgard, Duke University. INS-1 and INS-1 832/13 cells were maintained in RPMI 1640 medium (Gibco) supplemented with 1 mM sodium pyruvate, 50 µM 2-mercaptoethanol, 10 mM HEPES, 100 units/ml penicillin, 100 µg/ml streptomycin and 10% (v/v) heat-inactivated fetal bovine serum (Gibco), at 37°C with 5% CO_2_. Cultures were maintained to 70–80% confluence before being used in the transfection experiments.

INS-1 832/13 cells were seeded at a density of 1×10^6^ cells/well in antibiotic-free medium containing 5.5 mM glucose in 6-well plates for 4 days prior to transfection. Cells were transfected with mixtures containing 5 µg of Lipofectamine 2000 transfection reagent (Invitrogen), 1 pmole of a firefly luciferase reporter construct and 2 µg of pRSV-βgal plasmid encoding *E. coli* β-galactosidase in Optimem I-reduced serum medium. The transfected cells were maintained in this medium for 24 h before it was replaced with RPMI medium containing either 5.5 mM or 25 mM glucose for the next 24 h.

For transactivation assays, 1.5 µg of the luciferase reporter construct, 1.5 µg of plasmid overexpressing USF1, USF2, Sp1 (pSp1), Sp3 (pSp3) or pcDNA3 (empty vector) and 2 µg of pRSV-β-gal were mixed with 5 µg of Lipofectamine 2000 (Invitrogen) in Optimem I-reduced serum medium and transfected to INS-1 832/13 as described above. The luciferase and β-galactosidase assays were performed as described previously [21]. The luciferase activity was normalized with the β-galactosidase activity and presented as the relative luciferase activity.

### siRNA transfection

INS-1 832/13 cells were transfected with Sp1, USF1, USF2 and ChREBP siRNAs. In brief, 5×10^5^ INS-1 832/13 cells were plated in 6-well plates 24 h before transfection in Optimem I-reduced serum medium before transfected with with 25 ng of siRNAs targeted to rat Sp1, USF1, USF2, ChREBP or scrambled siRNA (Ambion) and 2 µg of lipofectamine 2000 in the presence of 2 ml growth medium for 5 min. The transfected cells were maintained at 37°C with 5% CO_2_ for 48 h before being harvested for RNA extraction and real time PCR analysis.

### Electrophoretic mobility shift assay (EMSA)

Nuclear extracts from INS-1 832/13 cell were prepared as described previously [21]. EMSA was performed using a non-radioactive EMSA. The two complementary oligonucleotides with their 3′-end labeled with biotin (BioBasic, Canada) were annealed and subjected to binding assays as described previously [31]. Supershift assays were performed by pre-incubating 2 µg of polyclonal antibodies against USF1 (sc-22), USF2 (sc-862), ChREBP (sc-21189), Sp1 (sc-59) or Sp3 (sc-644-G) [Santa Cruz Biotechnology] with nuclear extracts for 10 min before binding reactions were performed. The DNA-protein complexes were separated by 5% native polyacrylamide gel electrophoresis using 0.5% TBE. The DNA-protein complexes were transferred onto Biodyne membrane (PALL) and the bands were detected using the Lightshift Chemiluminescent EMSA kit (Pierce). The sequences of oligonucleotides used in these experiments are shown in Table 1.

**Table 1:** Oligonucleotides used in this study. Underline indicates mutated nucleotides.

Oligonucleotide	Sequence	Used for
pGL-P2 ΔKpnl Mu1F	5′ TCTCTATCGATA-TGCCCTTAGCTA 3′	Δ1 Mutant
pGL-P2 ΔKpnl Mu1R	5′ TAGCTAAGGGCA-TATCGATAGAGA 3′	
pGL-P2 ΔKpnl Mu2F	5′ TCTAATTCCTCG-GACCTCTTCTGT 3′	Δ2 Mutant
pGL-P2 ΔKpnl Mu2R	5′ ACAGAAGAGGTC-CGAGGAATTAGA 3′	
pGL-P2 ΔKpnl Mu3F	5′ CGTTTCTCCTGC-TACGTGCATCTG 3′	Δ3 Mutant
pGL-P2 ΔKpnl Mu3R	5′ CAGATGCACGTA-GCAGGAGAAACG 3′	
pGL-P2 ΔKpnl Mu4F	5′ TCTGCTAAAGAG-AACCCCCGTGCA 3′	Δ4 Mutant
pGL-P2 ΔKpnl Mu4R	5′ TGCACGGGGGTT-CTCTTTAGCAGA 3′	
pGL-P2 ΔKpnl Mu5F	5′ CAGCACCGCTCC-CGGTTTGAAGAG 3′	Δ5 Mutant
pGL-P2 ΔKpnl Mu5R	5′ CTCTTCAAACCG-GGAGCGGTGCTG 3′	
pGL-P2 ΔKpnl Mu6F	5′ CTGTTATGGTTG-CTCGAGTGAATG 3′	Δ6 Mutant
pGL-P2 ΔKpnl Mu6R	5′ CATTCACTCGAG-CAACCATAACAG 3′	
(−465/−460)mut_F	5′ TAAAGAGTACGTG**g** A**at**T**c**GCAGCACCGCTCC 3′	MuE1 mutant
(−465/−460)mut_R	5′ GGAGCGGTGCTGC**g** A**at**T**c**CACGTACTCTTTA 3′	
(−442/−437)mut_F	5′ CACCGCTCCAACC**gaat** T**c**CATCTGTTATGG 3′	MuE2 mutant
(−442/−437)mut_R	5′ CCATAACAGATG**g** A**attc**GGTTGGAGCGGTG 3′	
(−436/−431)mut_F	5′ TCCAACCCCCGTG**g** A**at**T**c**TTATGGTTGCGGT 3′	MuE3 mutant
(−436/−431)mut_R	5′ ACCGCAACCATAA**g** A**at**T**c**CACGGGGGTTGGA 3′	
(−408/−403)mut_F	5′ GGTTTGAAGAGA**g** A**at**T**c**CTACTCGA**t**T**c**AATGAATTGC 3′	MuE4 mutant
(−408/−403)mut_R	5′ CTGCAATTCATT**g** A**a**TCGAGTAG**g** A**at**T**c**TCTCTTCA 3′	
USF2CDS-F	5′ AAGCTTATGGACATGCTGGACCCGGGTCTG 3′	USF2 cloning
USF2CDS-R	5′ GGTACCTCACTGCCGGGTGCTCTCGCCC 3′	
USF1CDS-F	5′ AAGCTTATGAAGGGGCAGCAGAAAACAGC 3′	USF1 cloning
USF1CDS-R	5′ GTACCTTAGTTGCTGTCATTCTTGATGAC 3′	
M4 (+)	5′ GCTAAAGAGTACGTGCATCTGGCAGCACCG 3′	EMSA
M4 (−)	5′ CGGTGCTGCCAGATGCACGTACTCTTTAGC 3′	
M5 (+)	5′ CCAACCCCCGTGCATCTGTTATGGTTGCGG	EMSA
M5 (−)	5′ CCGCAACCATAACAGATGCACGGGGGTTGG 3′	
M5pmut E-box2 (+)	5′ CCAACCGAATTCCATCTGTTATGGTTGCGG 3′	EMSA
M5pmut E-box2 (−)	5′ CCGCAACCATAACAGATGGAATTCGGTTGG 3′	
M4+M5 (+)	5′ CATCTGGCAGCACCGCTCCAACCCCCGTGCATCTG 3′	EMSA
M4+M5 (−)	5′ CAGATGCACGGGGGTTGGAGCGGTGCTGCCAGATG 3′	
M4/5pmut (+)	5′ CATCTGGCAGCAGAATTCCAACC**gaat** T**c**CATCTG	EMSA
M4/5pmut (−)	5′ CAGATG**g** A**attc**GGTTGGAGCGGTGCTGCCAGATG 3′	

### Chromatin immunoprecipitation assay (ChIP)

INS-1 832/13 cells were seeded at a density of 6×10^6^ cells in a 100 mm dish and maintained in RPMI medium containing 5.5 mM glucose for 4 days. The cells were cultured in RPMI medium containing 5.5 mM or 25 mM glucose for 12 h. DNA and proteins were cross-linked by adding formaldehyde to the culture medium to the final concentration of 1% (v/v) at 37°C for 5 min. ChIP was performed as described previously [21] except that the pre-cleared chromatin was precipitated with 1 µg of anti-USF1, anti-USF2, anti-Sp1, anti-Sp3 or anti-ChREBP as described above. The transcription factor-bound DNA was amplified using Q-PCR with FastStart Universal SYBR Green master mix (Roche). Input (unbound DNA) of each group (5.5 or 25 mM glucose) was used to normalize the target DNA (set as 100%) and non-IgG condition was used as the reference in real time PCR. The primers used for amplifying the target site are −408 F/R (5′-GCGACCTCTTCTGTATCTGCTAA-3′ and 5′-AGACCTTCTGATTGGTGAAGAGG-3′) which flanked the Sp1 and USF sites of rat PC promoter [19], and Ex2 F/R primers (5′-GCCCATCAAGAAAGTAATGGTA-3′ and 5′-CTTGGCCACCTTAATGATGTCT-3′) that are located within exon 2 of the rat PC gene [27].

### Quantitative RT-PCR (Q-PCR)

Total RNA was isolated from cells using TRI Reagent (Molecular Research Center) and its concentration was determined by spectrophotometry. cDNA synthesis was carried out in a 20 µl-reaction mixture containing 500 ng of total RNA, 200 ng of random hexamers (Promega), 1x first-strand buffer (50 mM Tris-HCl, pH 8.3, 75 mM KCl, 3 mM MgCl_2_), 0.1 mM DTT, 1 mM each of dNTP and 200 units of SuperscriptIII reverse transcriptase (Invitrogen). Quantitation of PC expression was performed by real time PCR (Q-PCR) using *Taq*man probe. The Q-PCR was performed in a 12 µl-reaction mixture containing 1x *Taq*man PCR master mix (Roche), 2 µl of 1∶10 diluted cDNA, 1 µM of each primer and 0.5 µM of *Taq*man probe. The thermal cycling consisted of an initial incubation at 50°C for 2 min and 95°C for 10 min followed by 40 cycles of denaturation at 95°C for 15 s and annealing/extension at 60°C for 1 min using MxPro 3005 (Agilent Technologies). Expression of PC was normalized with that of 18 s rRNA and the results are shown as “relative gene expression”. The sequences of primers and probes for PC mRNA and 18 s rRNA gene are the same as previously described [19]. Q-PCR for the detection of USF1, USF2, Sp1 and ChREBP expression in INS-1 832/13 was performed using Sybergreen. The primers used for detection of rat USF1 (PPR45083A), USF2 (PPR49799A), Sp1 (PPR49794A) and ChREBP (PPR49636B) were purchased from Qiagen.

### Western blot analysis

Forty micrograms of nuclear and cytosolic extracts from INS-1 832/13 cells were subjected to 10% discontinuous SDS-PAGE before transferring onto PVDF membrane (PALL). The blots were incubated with appropriate dilution of anti-Sp1 (sc-59), anti-USF1 (sc-22), anti-USF2 (sc-862), anti-ChREBP (sc-21189) [Santa Cruz Biotechnology], anti-phospho Sp1 (ab59257), anti-tubulin (ab6046) or anti-laminB (ab16048) [Abcam], in 5% BSA in TBS-T overnight. The blots were washed with TBS-T before incubating with appropriate secondary antibodies conjugated with HRP for 3 h. The immunoreactive bands were visualized by an enhanced chemiluminescence using ECL Western substrate kit (Pierce).

### Statistical Analysis

All data are presented as the means ± SD from three independent experiments. Statistical analyses were performed using the ANOVA test. A P value<0.05 was considered to be statistically significant.

## Results

### Glucose induces PC mRNA expression in a time-dependent manner

**Figure 1:**
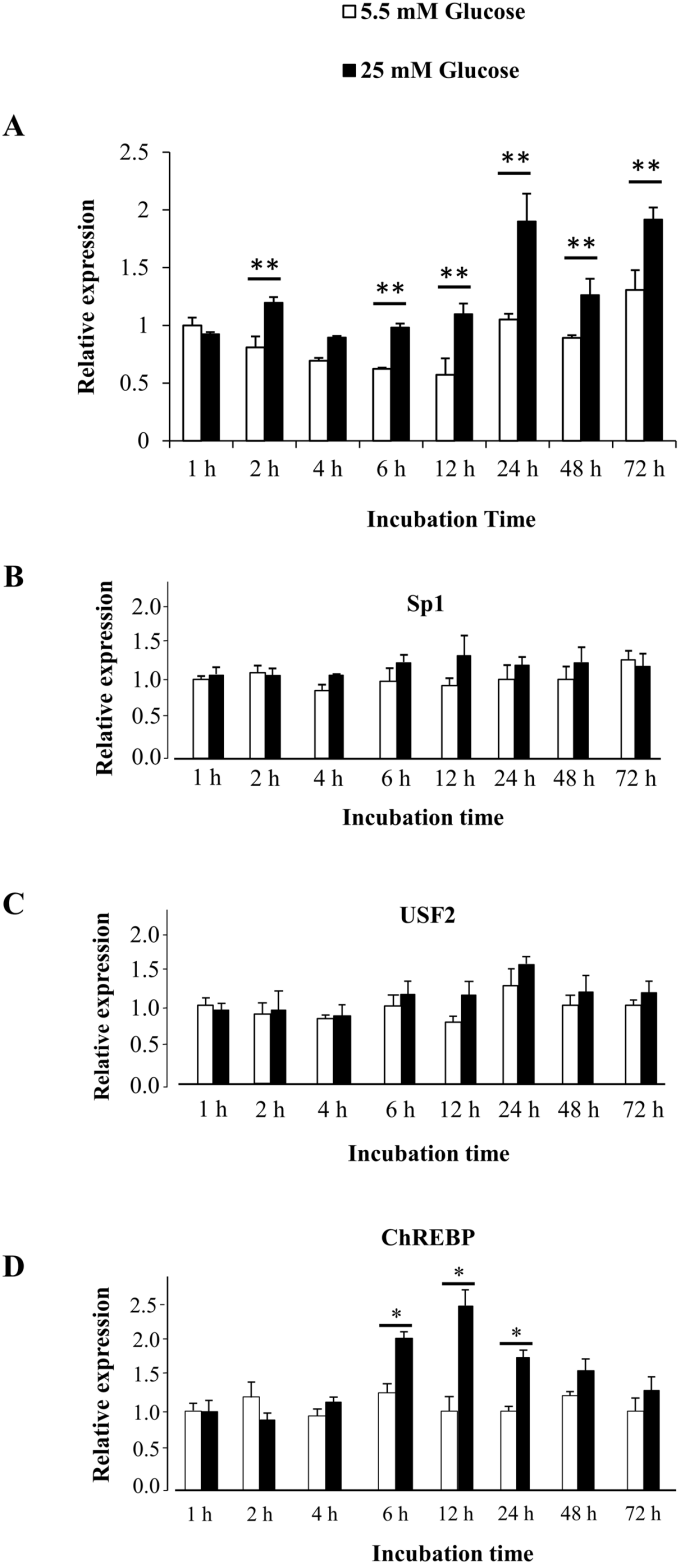
Glucose increases expression of PC and ChREBP mRNAs in INS-1 832/13 cells. INS-1 832/13 cells were cultured in RPMI medium containing normal (5.5 mM) or high (25 mM) glucose for 1, 2, 4, 6, 12, 24, 48 and 72 h. Total RNA was prepared from cells harvested at the indicated time points, converted to cDNA and analyzed by real time PCR. The expression of PC (A), Sp1 (B), USF2 (C) and ChREBP (D) was normalized with that of 18 s rRNA and shown as relative PC expression. Relative expression values of each gene obtained from cells maintained in RPMI containing 5.5 mM and 25 mM glucose at various time points were compared with those obtained from cells cultured in RPMI containing 5.5 mM at 1 h, which was arbitrarily set as 1. The statistical analysis was conducted using ANNOVA test where *P<0.05; **P<0.01.

Previous work has shown that incubation of rat islets with elevated concentrations of glucose resulted in the up-regulation of PC protein [9]. To examine whether the increased PC protein was due to up-regulation of PC mRNA, INS-1 cells were exposed to glucose at normal (5.5 mM) and high (25 mM) concentrations, followed by quantitative real time PCR analysis of PC mRNA expression. As shown in Figure 1A, incubation of the INS-1 cells with a high concentration of glucose resulted in a significant increase in PC mRNA expression at 2 h, 6 h, 12 h, 24 h, 48 h and 72 h (P<0.01).

### Multiple E-boxes in the distal promoter of rat PC gene function as the glucose-responsive element

**Figure 2:**
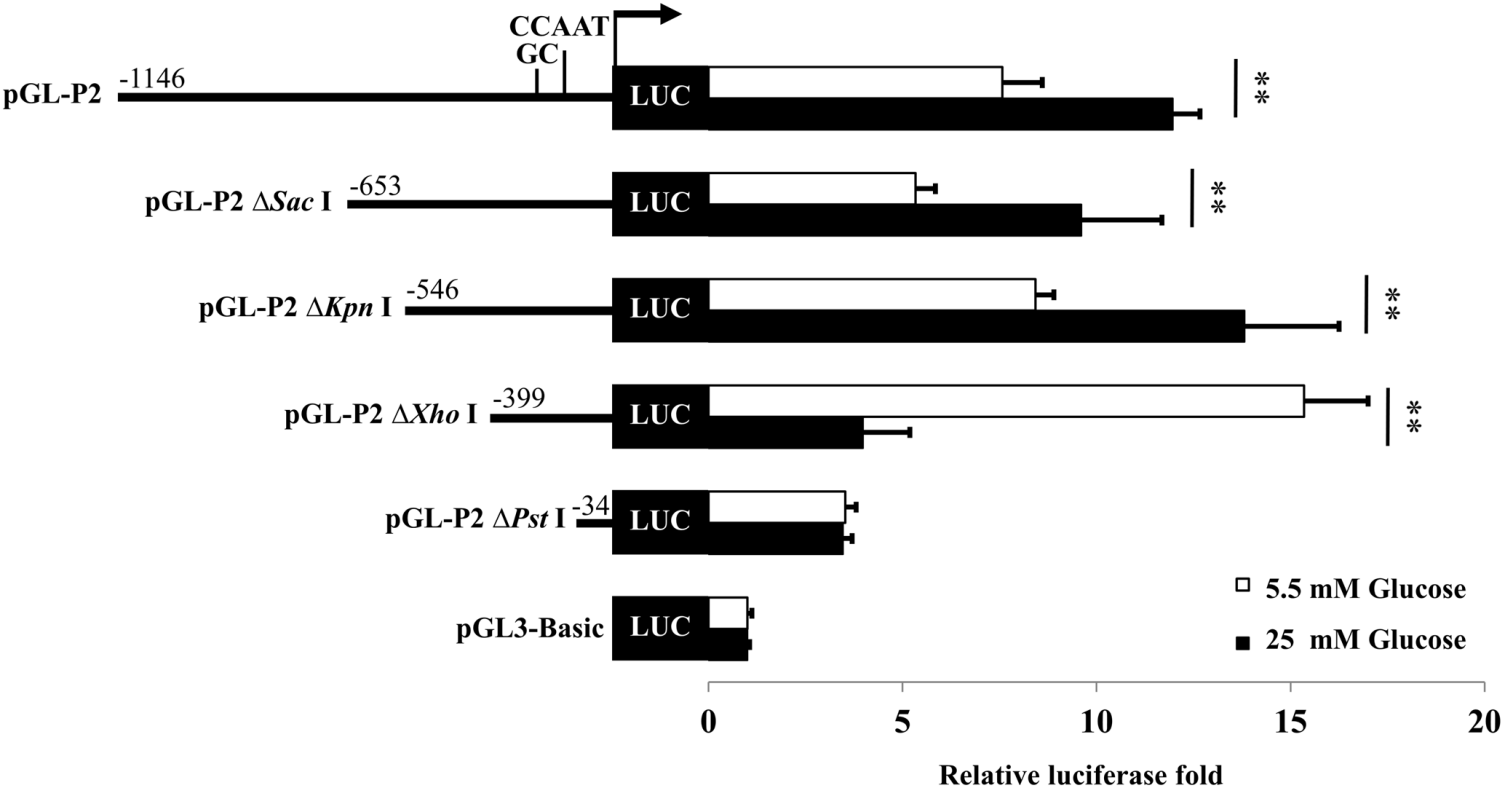
The P2 promoter of rat PC gene contains glucose-responsive element(s) (GRE). A series of 5′-truncated P2-luciferase reporter constructs were transfected into INS-1 832/13 cells. The transfected cells were maintained in RPMI containing normal (5.5 mM) or high (25 mM) glucose for 24 h. The luciferase activity of each construct was normalized to the β-galactosidase activity and expressed as relative luciferase activity. Relative luciferase values obtained from transfected cells maintaining in high glucose medium were presented as fold change relative to those maintaining in normal concentration of glucose, each of which was arbitrarily set as 1. The statistical analysis was conducted using ANOVA test where **P<0.01.

Because the distal promoter of the rat PC gene is the only promoter that is transcriptionally active in rat pancreatic β-cells [17] and mostly active in the insulinoma cell line, INS-1 832/13 [26] we examined whether the distal promoter of the PC gene contains a glucose-responsive element (GRE) by transfecting the 1.2 kb distal promoter-luciferase chimeric reporter constructs into the INS-1 832/13, a cell line that responds to glucose more robust than the INS-1 cell line [30]. This promoter fragment has previously been characterized to contain full basal and tissue-specific *cis*-acting elements [21]. The transfected cells were maintained in the medium containing 5.5 mM or 25 mM glucose for 24 h. As shown in Figure 2, the transfected cells maintained in medium containing 25 mM glucose possessed luciferase activity approximately 2-fold higher than those maintained at 5.5 mM glucose, suggesting that glucose exerts its stimulatory effect on PC expression via GRE located within the 1.2 kb distal promoter of PC gene. To define the GRE, we transfected a series of 5′-truncated distal promoter-luciferase reporter constructs into INS-1 832/13 cells. Truncations from nucleotides −1146 to −653 (pGL-P2ΔSacI construct) or to nucleotide −546 (pGL-P2ΔKpnI) did not affect glucose-mediated transcriptional activation of the luciferase reporter gene (Figure 2). However, truncation to nucleotide −399 (pGL-P2ΔXhoI) not only completely eliminated the glucose induction effect but this also unexpectedly increased the luciferase reporter gene under low glucose conditions. Further truncation of the P2 promoter to the −34 region (pGL-P2ΔPstI), yielded a similar result with that of the pGL-P2ΔXhoI construct, suggesting that the GRE was located between nucleotides −546 and −399.

**Figure 3:**
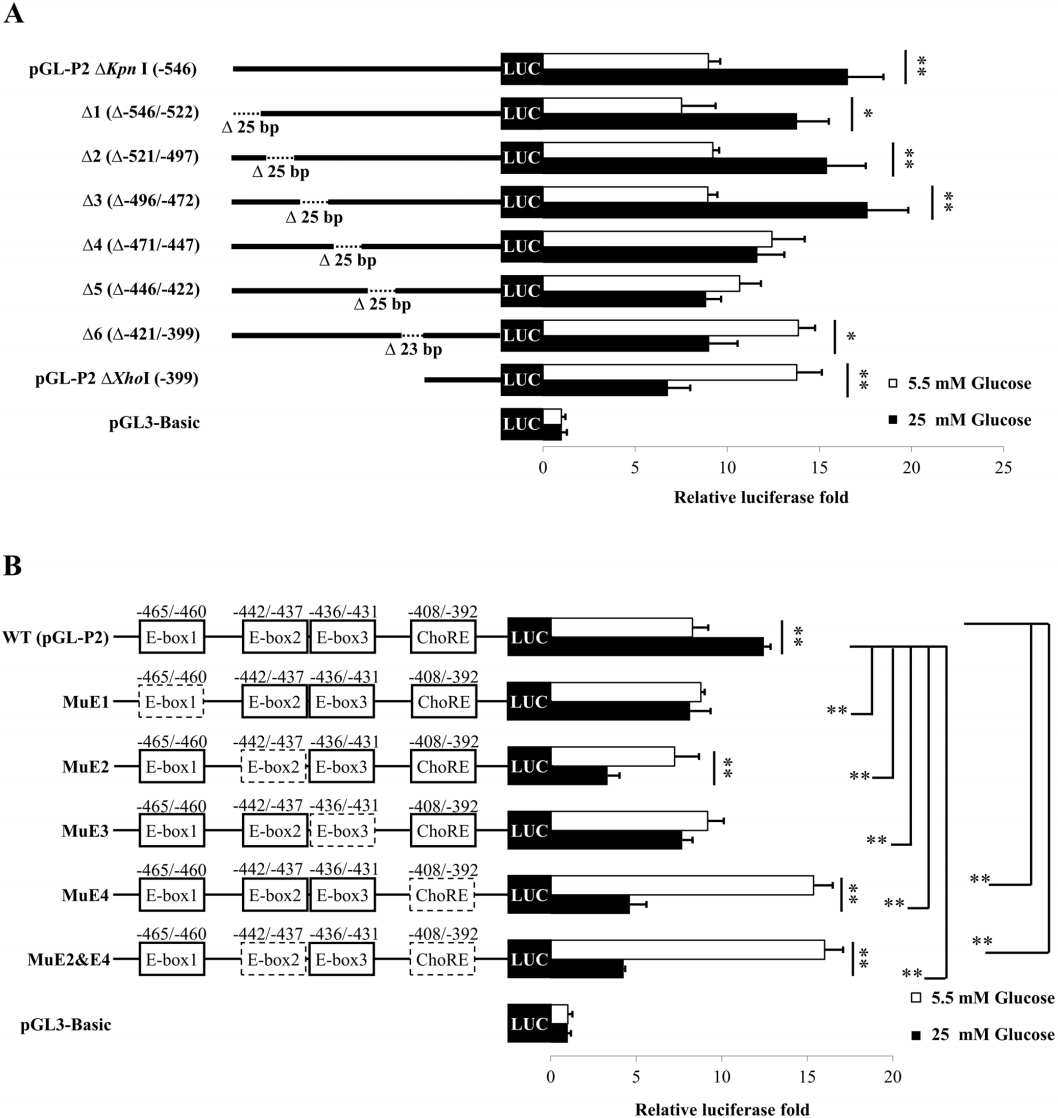
Multiple E-boxes in the P2 promoter of the PC gene function as GREs. **A**, A series of 25 or 23-nucleotide internal deletions were generated across the −546 to −399 of P2 promoter (Δ1, Δ2, Δ3, Δ4, Δ5 and Δ6). **B**, The WT P2 promoter construct containing mutation at E1, E2, E3 and whole ChoRE (MuE1, MuE2, MuE3, MuE4 and MuE2&E4) were generated and transiently transfected into INS-1 832/13 cells. The transfected cells were maintained in RPMI containing normal (5.5 mM) or high (25 mM) glucose for 24 h. The luciferase activity of each construct was normalized to the β-galactosidase activity and expressed as relative luciferase activity. Relative luciferase values obtained from transfected cells maintaining in high glucose medium were presented as fold change relative to those obtained from those maintaining in normal concentration of glucose, each of which was arbitrarily set as 1. The statistical analysis was conducted using ANOVA test where *P<0.05; **P<0.01.

**Figure 4:**
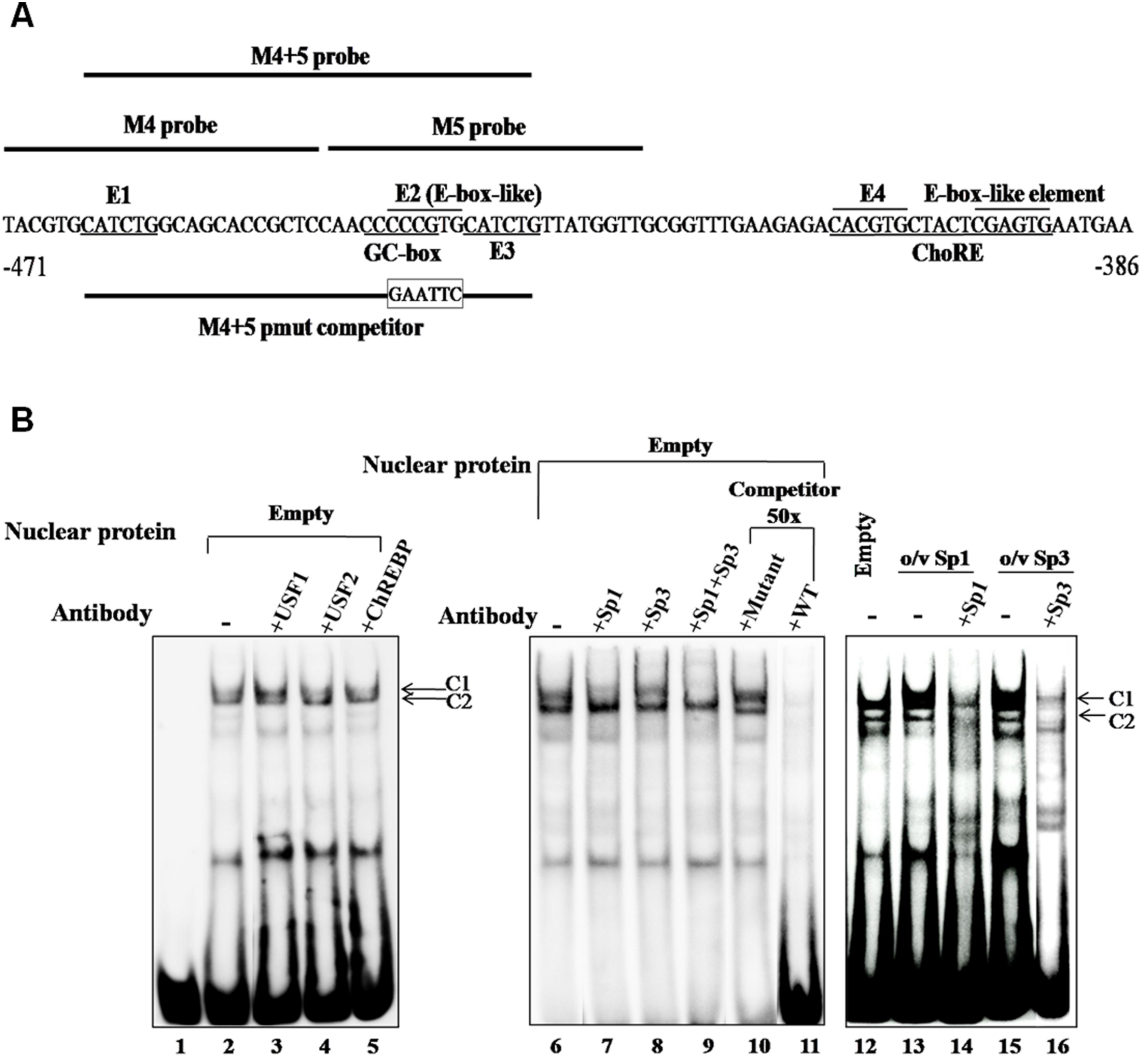
Sp1 and Sp3 bind E-box-like element (E2) *in vitro*. **A**, Nucleotide sequence of M4+M5 and location of various E-boxes [E1, E2 (E-box like), E3 and E4]. **B**, M4+M5 probe was incubated with nuclear extract of INS-1 832/13 cells and subjected to EMSA. Lanes 1, M4+5 probe alone; lanes 2 and 6, probe incubated with nuclear extract; lanes 3–5, nuclear extracts were pre-incubated with anti-USF1, anti-USF2, anti-ChREBP, respectively. Lanes 7–9, nuclear extracts incubated with anti-Sp1, anti-Sp3 antibodies or both before the probes were added into the reaction, respectively. Lanes 10–11, nuclear extracts were pre-incubated with 50-fold excess mutant or wild type unlabeled oligonucleotide before the probes were added into the reaction, respectively. Lane 12, probe incubated in nuclear extract of INS-1 832/13 cells transfected with an empty vector (Empty) or nuclear extract of INS-1 832/13 cells transfected with plasmid over-expressing Sp1 (o/v Sp1) (lane 13) or Sp3 (o/v Sp5) (lane 15). Lanes 14 and 16, nuclear extracts of INS-1 832/13 cells over-expressing Sp1 or Sp3 were pre-incubated with anti-Sp1 or anti-Sp3 antibody, respectively before probe was added into the reaction. Arrows (C1, C2) represent DNA-protein complexes.

To precisely localize the GRE, we generated a series of internal deletions across the −546 to −399 region and the resulting constructs [Δ1(−546/−522), Δ2(−521/−497), Δ3−496/−472), Δ4(−471/−447), Δ5(−446/−422) and Δ6(−421/−399)] were transfected into INS-1 832/13 cells. As shown in Figure 3A, deletion of the nucleotides −546/−522 (Δ1), −521/−497 (Δ2) and −496/−472 (Δ3) did not affect the glucose response, however deletion of the nucleotides −471/−447 (Δ4) resulted in a complete loss of the glucose response. Similar results were observed when the nucleotides −446/−422 (Δ5) and −421/−399 (Δ6) were deleted. Of particular interest, the deleted nucleotides in Δ4, Δ5 and Δ6 mutant constructs corresponded to the three copies of canonical E-boxes (CANNTG), designated E1 (−465/−460), E3 (−436/−431) and E4 (−408/−403), and one E-box-like element [E2 (−442/−437), respectively (See Figure 3A). E-boxes have previously been reported to be involved in many glucose-responsive genes [32]–[37]. To examine whether these four E-boxes confer glucose-mediated transcription induction of the PC gene, each of them was mutated. As shown in Figure 3B, mutations of E1, E2 or E3, resulted in a complete loss of glucose-mediated transcription induction of the reporter gene but had no effect on transcriptional induction under normal glucose (5.5 mM). In contrast, mutation of a whole ChoRE consisting of E4 and E-box-like (see ChoRE in Figure 4A) not only eliminated high glucose induction effect but also resulted in a 2-fold increase in the reporter activity under low glucose condition. This result was in agreement with those of the pGL-P2ΔXhoI (Figure 2) and Δ6 mutants (Figure 3A), suggesting ChoRE functions as an activator under high glucose induction condition and as a repressor under low glucose condition.

### Sp1 functions as a glucose-responsive transcription factor binding to E-box like element

To identify which transcription factors might bind to these four E-boxes, we performed EMSA using two double stranded oligonucleotide probes corresponding to various E-boxes. Incubation of M4+M5 probe which covers E1, E2 and E3 (Figure 4A) with nuclear extract of INS-1 832/13 cells produced two prominent species of DNA-protein complexes (C1 and C2, respectively) (Figure 4B, lane 2) compared to control with no nuclear extract (lane 1). Since the transcription factors, USF1, USF2 and ChREBP have been reported to regulate several glucose responsive genes via binding to E-boxes in their enhancer regions, we performed a supershift assay using antibodies against these three transcription factors. As shown in Figure 4B, neither of these antibodies affected the formation of both C1 and C2 complexes, indicating that these two complexes could not be attributed to the above three transcription factors (lanes 3–5, respectively). Re-examination of the nucleotide sequence surrounding these E-boxes by the PROMO transcription factor binding site database [38] revealed the presence of CCCCCG (positions −444/−439), within E2. This sequence is similar to the GC-rich, a putative binding site of Sp1 and Sp3 transcription factors [39]. We examined if this is the case by incubating the binding reaction in the presence of anti-Sp1 or anti-Sp3 antibody. Addition of anti-Sp1, anti-Sp3 or both antibodies reduced the formation of C1 complex (lanes 7–9, respectively). Incubation of the probe with nuclear extract of INS-1 832/13 overexpressing Sp1 or Sp3 produced a predominant strong band of C1 (lanes 13 and 15, respectively) which was much stronger than that observed from nuclear extract of cells transfected with an empty vector (lane 12). Incubation of anti-Sp1 or anti-Sp3 antibody to the nuclear extract of the Sp1- or Sp3-overexpressing cells prior to the reaction markedly eliminated this strong band (lanes 14 and 16, respectively), further confirming that Sp1 and Sp3 bind to this CCCCCG within E2. To examine whether the CCCCCG motif indeed mediates C1 and C2 formation, we performed a competition EMSA in which the competitor sequence lacking this sequence (M4+5 pmut competitor) was used in the assay. In contrast to the use of WT sequence as a competitor which can eliminate the complex formation (lane 11), the competitor lacking CCCCCG sequence failed to prevent the complex formation (lane 10), suggesting that Sp1 and Sp3 bind to this CCCCCG within E2.

**Figure 5:**
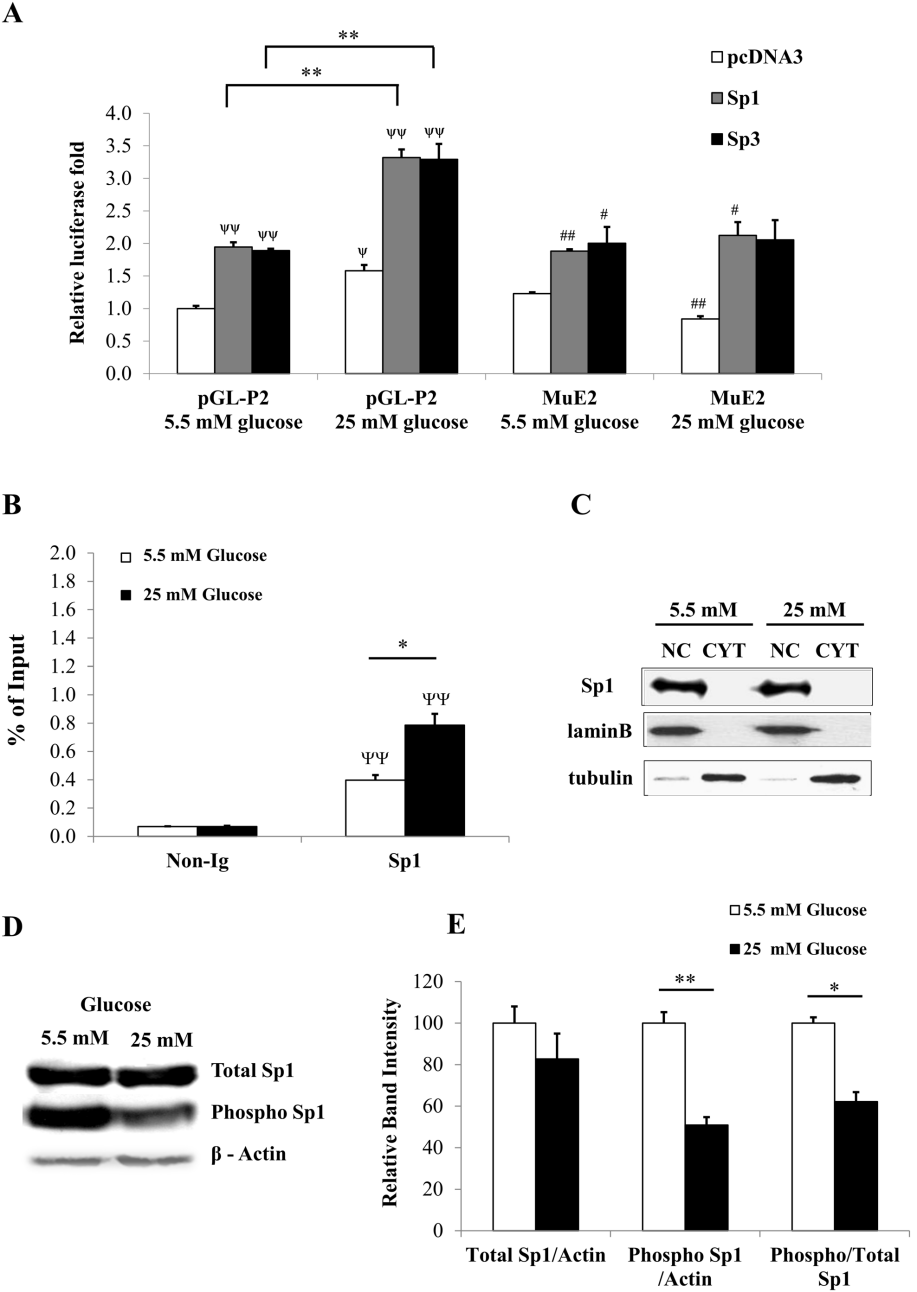
Sp1 regulates glucose-induced PC expression through E-box-like element (E2) in P2 promoter of the PC gene. **A**, The WT-P2(pGL-P2) or its mutation at E2-luciferase reporter constructs (MuE2) with plasmid overexpressing Sp1, Sp3 or empty vector (pcDNA) were transfected into INS-1 832/13 cells before they were maintained in medium containing low (5.5 mM) or high (25 mM) glucose before their luciferase activities were assayed. Relative luciferase activity of cells co-transfected with pGL-P2 (WT) or mutant promoter construct with empty vector (pcDNA), pSp1 or pSp3 maintained at 5.5 mM or 25 mM were normalized with that of cells co-transfected with pGL-P2 and empty vector maintained at 5.5 mM, which was arbitrarily set as 1. The statistical analysis was conducted using ANOVA test. Ψ (P<0.05) and ΨΨ (P<0.01), compared with cells transfected with pGL-P2 and pcDNA maintained at 5.5 mM glucose. # (P<0.05) and ## (P<0.01), compared with cells transfected with MuE2 maintained at 5.5 mM glucose. **P<0.01 (transactivation of WT promoter by Sp1 or Sp3 under 5.5 mM and 25 mM glucose). **B**, The Sp1-bound chromatin was prepared from INS-1 832/13 cells grown in low (5.5 mM) or high (25 mM) glucose, fragmented and immunoprecipitated with anti-Sp1 antibody and subjected to real time PCR. The fluorescence signals obtained from quantitation of immunoprecipitated fraction was normalized to the input levels. The input fraction was the sonicated Sp1-bound DNA before immunopreripitating with anti-Sp1 antibody. The statistical analysis was conducted by ANOVA test where *,ΨΨ P<0.01. **C**, Western blot analysis of nuclear (NC) and cysolic (CYT) extracts of INS-1 832/13 cells maintained under 5.5 or 25 mM glucose with anti-Sp1 antibody. Loading controls of the cytosolic and nuclear proteins were assessed by stripping the blot and re-probed with anti-tubulin and anti-lamin B antibody, respectively. **D**, A representative of Western blot analysis of nuclear extracts of INS-1 832/13 cells maintained in the presence of 5.5 or 25 m glucose with anti-Sp1, anti-phospho Sp1. Control loading was assessed by stripping the blot and re-probed with anti-actin antibody. E, The intensity of total Sp1, phospho-Sp1 bands was quantitated and normalized with that of actin band and shown as the ratios of total Sp1/actin, phospho-Sp1/actin and total Sp1/phospho-Sp1 from three independent experiments. The statistical analysis was conducted using ANOVA test where *P<0.05 **P<0.01.

To examine whether over-expression of Sp1 or Sp3 in INS-1 832/13 could influence PC promoter activity, we performed a reporter assay in which we co-transfected the WT PC promoter-reporter construct or promoter-reporter construct containing mutated CCCCCG with plasmids over-expressing Sp1 or Sp3 in the INS-1 832/13 and cultured the transfected cells in the medium containing normal (5.5 mM) or high (25 mM) of glucose. As shown in Figure 5A, in the presence of 5.5 mM glucose, over-expression of Sp1 or Sp3 increased the reporter activity of the WT promoter construct approximately 2-fold while in the presence of 25 mM glucose, over-expression of Sp1 or Sp3 further increased the reporter activity of the WT promoter to 5-fold. In the mutant construct, in the presence of 5.5 mM, over-expression of Sp1 or Sp3 was capable of increasing the reporter activity to the similar levels as that of the WT construct. However, further increase of reporter activity of this mutant construct mediated by Sp1 or Sp3 was lost, indicating that mutation of CCCCCG sequence blunts Sp1- and Sp3-mediated transactivation under high glucose concentration. We next confirmed the above result by performing a ChIP assay that showed *in situ* binding of Sp1 and Sp3 to CCCCCG in the P2 promoter of the rat PC gene in INS-1 832/13 cells maintained in medium containing either 5.5 mM or 25 mM glucose. As shown in Figure 5B, in the presence of 25 mM glucose, Sp1 appeared to bind to the CCCCCG sequence approximately 2-fold greater than in the presence of 5.5 mM glucose. Although we have shown that Sp3 also bound to E2 by EMSA, we were not able to show that Sp3 bound to E2 in the presence of either 5.5 mM or 25 mM glucose using the ChIP assay. This may suggest that Sp1 rather than Sp3 regulates PC expression via CCCCCG sequence *in vivo*. This enhanced binding of Sp1 to CCCCCG sequence in the presence of high glucose cannot be attributed to an increased level of Sp1 mRNA as its mRNA expression did not change after cells were exposed to high glucose from 1 h to72 h. (Figure 1B). Likewise, the increased binding of Sp1 was not due to the increased entry rate of Sp1 into nucleus because the amounts of Sp1 detected by Western blot analysis in the nucleus and cytoplasm of cells maintained in the presence of 5.5 mM and 25 mM glucose at the time when ChIP was performed (12 h) were no difference from one another (Figure 5C). Furthermore, most of the Sp1 was also found in the nucleus. Several reports have demonstrated that phosphorylation/dephosphorylation of Sp1 in response to elevated glucose levels contributes to its transcriptional activity [40]. We detected total and phosphorylated Sp1 in INS-1 832/13 cells cultured in the medium containing low and high glucose. As shown in Figure 5D, INS-1 832/13 cells grown in the presence of 5.5 mM and 25 mM glucose expressed similar amounts of total Sp1. However, most Sp1 presence in the cells maintained under low glucose was detected in the phosphorylated form while only 50% of total Sp1 presence in cells maintained under high glucose was detected in the phosphorylated form (Figure 5E). It is very likely that the enhanced recruitment of Sp1 to CCCCCG sequence detected by the ChIP assay can be attributed to the dephosphorylation of this protein. The dephosphorylation form of Sp1 has been shown to be a transcriptionally active form which contributes to the transcriptional induction of the acetyl-CoA carboxylase 1 (ACC1) gene [41].

### USF2 preferentially binds to E1 while an unknown factor(s) binds to E3

**Figure 6:**
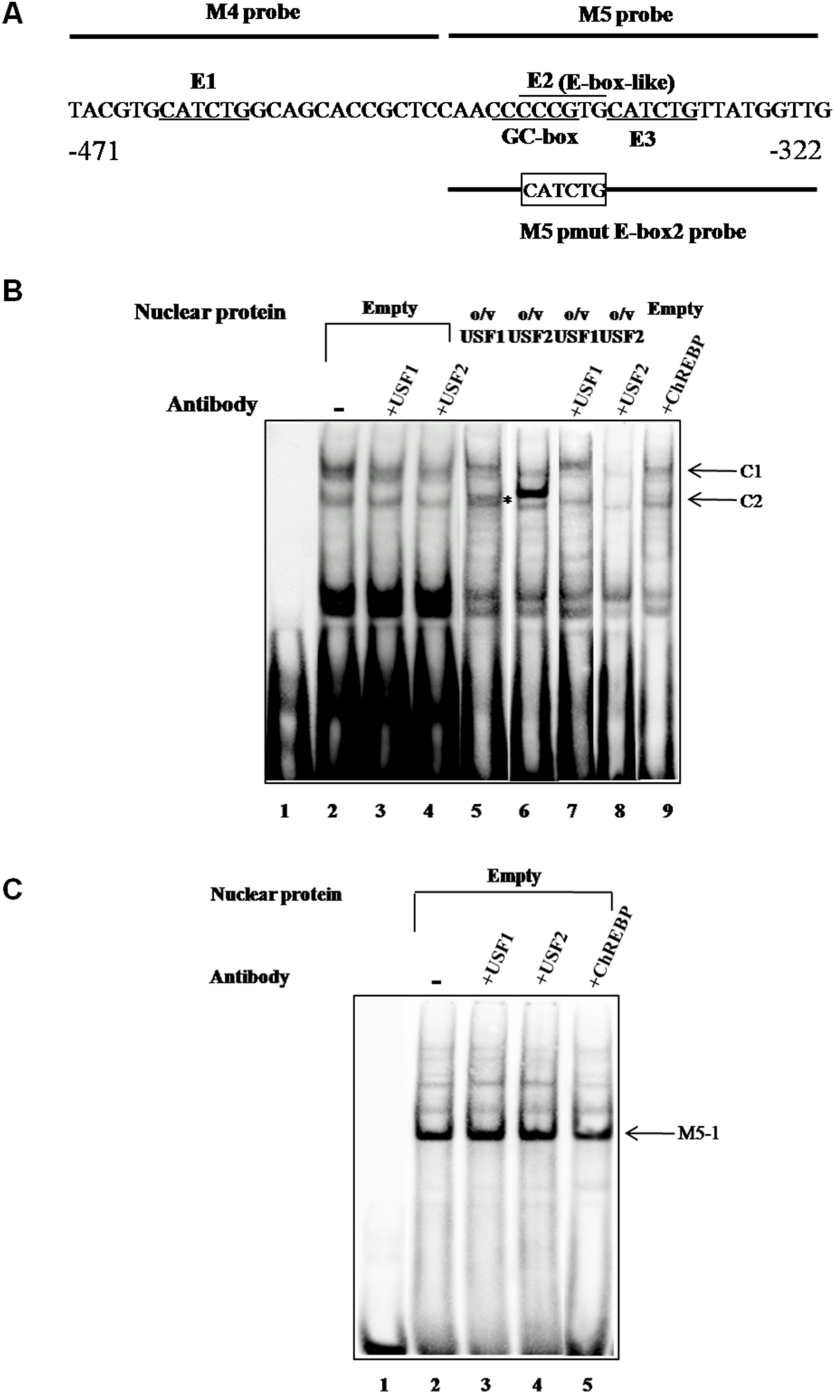
USF1 and USF2 bind to the E1 in the P2 promoter of the PC gene. **A.** Nucleotide sequences of M4, M5 and M5 pmut E2 probes. E-box and GC-box are underline. **B,** EMSA of M4 probe with INS-1 832/13 nuclear extract. Lane 1, M4 probe alone; lane 2, probe incubated with nuclear extract; lanes 3–4, nuclear extracts pre-incubated with anti-USF1 or anti-USF2 antibody before the probes were added into the reaction, respectively. Lanes 5–6, probe incubated with nuclear extract of INS-1 832/13 overexpressing USF1 or USF2, respectively. Lanes 7 and 8, nuclear extracts of INS-1 832/13 overexpressing USF1 pre-incubated with anti-USF1 antibody or overexpressing USF2 pre-incubated with anti-USF2 antibody before the probe was added into the reaction, respectively. Lane 9, INS-1 832/13 nuclear extract pre-incubated with anti-ChREBP antibody before the probe was added into the reaction. **C.** EMSA of M5 pmut E2 probe with an INS-1 832/13 nuclear extract. Lane 1, M5 pmut E2 probe alone; lane 2, probe incubated with nuclear extract; lanes 3–5, nuclear extracts pre-incubated with anti-USF1, anti-USF2 or anti-ChREBP antibody before the probes were added into the reaction, respectively. Arrows represent DNA-protein complexes.

Although the EMSA shown in Figure 4B suggested that USF1, USF2 and ChREBP did not bind to the probe, this may not be the only conclusion because E1 and E3 are located close to the extreme 5′- and 3′-ends of M4+M5 probe. This may restrict the efficiency of binding of (an) additional transcription factor(s), if any, apart from Sp1 and Sp3. To rule out this possibility, we synthesized the M4 probe which contains only E1 in the middle for EMSA analysis (Figure. 6A). Incubation of the M4 probe with a nuclear extract of INS-1 832/13 revealed weak DNA-protein complexes (data not shown), suggesting the poor binding of the candidate transcription factor(s) to this probe. However, a prolonged exposure (10 min) of the blot produced two weak DNA-protein complexes (C1 and C2) (Figure 6B, lane 2). Incubation of anti-USF1 or anti-USF2 antibody in the reaction marginally affected both complexes (lanes 3 and 4). However, incubation of M4 probe with the nuclear extract prepared from INS-1 832/13 cell overexpressing USF1 produced an additional weak band corresponding to the C2 complex (lane 5, asterisk) while incubation of the same probe with a nuclear extract of INS-1 832/13 cells overexpressing USF2 also produced an additional band corresponding to the C2 complex but was much stronger (lane 6). Furthermore incubation of anti-USF1 or anti-USF2 antibodies prevented C2 formation (lanes 7 and 8), suggesting that USF2 preferentially binds to E1. Although both anti-USF1 and USF2 antibodies prevented at least one of the two complexes, anti-ChREBP antibody did not affect formation any of these complexes (lane 9), suggesting that ChREBP did not bind to E1.

We also conducted EMSA using the new probe, M5 which contains mutated CCCCCG in E2 (M5 pmut E2 probe in Figure 6A), leaving only E1 intact in the middle (M5 pmut E-box2, Figure 6A). Any binding observed in this assay would result from binding of the nuclear factor to E3 only. Incubation of this probe with a nuclear extract of non-transfected INS-1 832/13 produced one prominent band, M5–1 (Figure 6C, lane 2). Incubation of the binding reaction in the presence of anti-USF1, anti-USF2 or anti-ChREBP (lanes 3–5, respectively) did not affect this complex, suggesting other nuclear factors rather than these three proteins bind to E3.

### Glucose enhances binding of USF2 and ChREBP to E4

**Figure 7:**
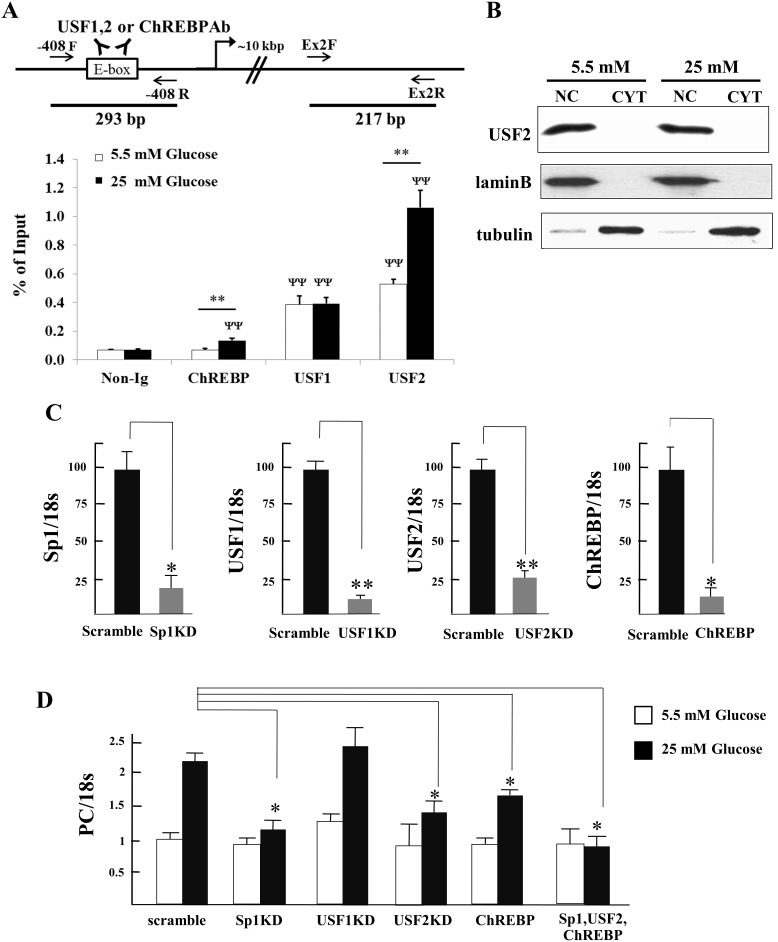
High glucose enhances binding of USF2 and ChREBP to E4 in the P2 promoter of the PC gene and suppression of Sp1, USF2 and ChREBP expression blunts glucose-induced expression of endogenous PC expression in INS-1 8322/13. **A**, *Top panel*, a schematic representation of the P2 promoter region with E-box4 and primer binding sites for quantitative real time PCR indicated. *Bottom panel*, the USF1-, USF2- or ChREBP-bound chromatin was prepared from INS-1 832/13 cells grown in low (5.5 mM) or high (25 mM) glucose was prepared, immunoprecipitated with their corresponding antibodies and subjected to real time PCR using the primers indicated above. The fluorescence signals obtained from the immunoprecipitated fractions were normalized to those obtained from the input fraction which was the sonicated transcription factor-bound DNA before immunoprecipitating with the antibodies. The statistical analysis was conducted by ANOVA test where **P<0.01 compared between low and high glucose concentrations. ^ΨΨ^P<0.001 compared with the fraction that was immunoprecipiated with no antibody at both low or high glucose concentration. **B**, Western blot analysis of nuclear (NC) and cysolic (CYT) extracts of INS-1 832/13 cells maintained under 5.5 or 25 mM glucose with anti- USF2 antibody. Loading controls of the cytosolic and nuclear proteins were assessed by stripping the blot and re-probed with anti-tubulin and anti-lamin B antibodies, respectively. **C**, INS-1 832/13 cells were mock- or transfected with siRNAs targeted to Sp1, USF1, USF2 and ChREBP. The transfected cells were cultured in the medium containing low (5.5 mM) or high (25 mM) glucose for the next 24 h before the expression of Sp1, USF1, USF2, ChREBP and PC mRNAs was measured by quantitative real time PCR and their expression levels were normalized with that of 18 s rRNA. The values obtained from scramble and each knocked down cells are expressed relative to that obtained from the mocked transfection, which was arbitrarily set as 100%. The values shown are means ± standard deviations of the three independent experiments (n = 3). The statistical analysis was conducted using ANOVA test where *P<0.05; **P<0.01.

We showed that E4 serves as a binding site for USF1 and USF2 [19], however the functional importance of this binding site with respect to the glucose-induction was unknown. Pedersen *et al*. [26] have previously shown that ChREBP binds relatively weakly to this site. To examine whether the recruitment of USF1, USF2 and ChREBP to this E-box is enhanced during high glucose induction, we performed a ChIP assay of this E4 with anti-USF1, anti-USF2 and anti-ChREBP antibodies in INS-1 832/13 cells maintained in the medium containing 5.5 mM and 25 mM glucose. As shown in Figure 7A, cells grown in high glucose medium did not affect the recruitment of USF1, instead recruitment of USF2 and ChREBP to E4 was increased (Figure 7A). The enhanced binding of USF2 to E4 in the presence of high glucose could not result from an increased level of USF2 mRNA expression as its abundance was unchanged after the cells were exposed to 25 mM glucose from 1 h to 72 h (Figure 1C). The increased binding of USF2 to E4 was not due to the increased entry rate of this transcription factor to nucleus because the amounts of USF2 in the nucleus of cells maintained under low and high glucose concentrations were similar (Figure 7B). Also most USF2 were found in the nucleus. We failed to detect expression of the ChREBP protein in INS-1 832/13 cells grown under 5.5 mM and 25 mM glucose by Western blot analysis, suggesting that the expression of ChREBP in INS-1 832/13 may be rather low compared to other types of cells, such as liver. This rather weak expression of endogenous ChREBP in INS-1 832/13 or rat islets was also seen in the previous report [26]. Nevertheless, the increased binding of ChREBP to E4 is partly contributed by increased ChREBP mRNA abundance. As shown in Figure 1D, expression of ChREBP mRNA was increased to 2–2.5-fold after INS-1 832/13 cells were exposed to 25 mM glucose for 6 h, 12 h and 24 h.

Finally we confirmed the functional role of Sp1, USF1, USF2 and ChREBP in regulating glucose-mediated transcriptional induction of PC expression. We suppressed expression of these four transcription factors by their respective siRNAs in INS-1 832/13 and maintained the transfected cells in the medium containing normal or high glucose before analyzing the expression of PC mRNA by real time PCR. As shown in Figure 7C, upon transfection of siRNAs targeted to Sp1 (Sp1 KD), USF1 (USF1 KD), USF2 (USF2 KD) or ChREBP (ChREBP KD) resulted in 80%, 90%, 75% and 80% reduction in expression of their respective mRNAs. As shown in Figure 7D, suppression of USF1 did not appear to affect glucose-induced PC mRNA expression while suppression of Sp1, USF2 or ChREBP expression resulted in 50%, 40% and 30% reduction of glucose-induced PC mRNA expression. Combined knockdown of USF2, Sp1 and ChREBP expression completely prevented glucose-induced PC mRNA expression (Figure 7D). These data suggest that the glucose-mediated transcriptional activation of PC in the P2 promoter is regulated by these three transcription factors, albeit their absolute degrees of the control are varied.

## Discussion

Pyruvate carboxylation has been shown to be a crucial anaplerotic reaction which regulates glucose-induced insulin secretion in pancreatic islets. PC catalyzes the above reaction and is highly abundant in this tissue where it participates in the pyruvate cycling, a process by which a coupling factor, NADPH is formed and required for glucose-induced insulin secretion (for review see [42], [43]). Suppression of PC expression impairs anaplerosis concomitant with the reduced glucose-induced insulin secretion [12], [13]. Previous work has shown that expression of PC in isolated rat islets is inducible by exogenous glucose and its expression is highly correlated with the flux toward pyruvate carboxylation [44]. Here we showed that induction of PC expression by glucose in the rat insulinoma cell line, INS-1 832/13 is rather slow, the levels of mRNA expression is increased to only 1.5-fold within the early hours before reaching maximum at 24 h. The slow induction of PC mRNA expression was also observed in the isolated rat islets maintained under high glucose containing medium [8]. It is possible that the allosteric regulation of PC by acetyl-CoA may be important mechanism to increase activity of PC during this early period before transcription of this gene is activated. It is well known that equal amounts of pyruvate enter pyruvate carboxylation by PC and pyruvate decarboxylation by PDH [6], [7], [9], [45]. The acetyl-CoA formed by PDH-catalyzed reaction can in turn allosterically activate PC activity during this early period.

In this report we identified a complex GRE within the P2 promoter of the rat PC gene. This GRE consists of four tandem E-boxes, locating at −465/−460 (E1), −442/−437 (E2), −436/−431(E3) and −408/−403 (E4) and as the glucose sensor. It is interesting to note that although these four E-boxes are well defined in the distal promoter of the rat PC gene, only E3 and E4 are also conserved in the distal promoter of the human PC gene, while the nucleotides corresponding to E1 and E2 contains one nucleotide difference in the distal promoter of the human PC gene [20]. This raises the possibility of subtle difference of transcriptional activation of PC in response to glucose between the rat and the human. Nevertheless, mutational analysis of these sequences of distal promoter of rat PC gene showed that mutating one of these E-boxes is sufficient to eliminate glucose-mediated transcriptional activation of P2 promoter activity, suggesting that these four E-boxes are equally important. It is noted that E1 and E3 resemble the canonical E-box (CANNTG) while E4 is part of the previously reported ChoRE [26]. While both endogenous USF1 and USF2 can bind E1, overexpression of USF1 or USF2 in INS-1 832/13 cells revealed that USF2 preferentially binds E1 much stronger than USF1. However, ChREBP did not bind this E-box, possibly because binding of ChREBP requires two adjacent E-boxes separated by 5 nucleotides [46]. Although the E3 sequence is similar to the E1 sequence, neither USF1 nor USF2 binds to E1, it appears to bind strongly to an unknown nuclear factor whose identity remains unidentified. We have previously shown that E4 serves as a binding site of USF1 and USF2 [21]. Although mutation of E4 moderately reduced P2 activity under basal conditions, its role in glucose-mediated transcriptional induction at that time was unknown [21]. Pedersen *et al* [26] previously reported that this E-box also serves as a binding site of ChREBP which mediates glucose-induced transcriptional induction of the P2 promoter. They have also shown that high glucose promotes the recruitment of ChREBP, but not USF1 or USF2, to this E-box. However, we have found that high glucose not only enhances binding of ChREBP, but also USF2, to this E-box. It is well known that USF2 can form a homodimer or a heterodimer with USF1, but not with ChREBP, to regulate transcription [47]. Likewise, ChREBP/Mlx heterodimer is required to activate glucose-dependent transcription. Although initial reports have clearly demonstrated that ChREBP transcriptional activity is closely associated with elevated concentrations of glucose [23], [48]–[49] via complicated post-translational modifications [24], [25], [50], growing evidence has now indicated that both USF1 and USF2 are capable of regulating transcription of several glucose-dependent genes [34], [51]–[53]. Although the expression levels of USF2 can be induced by elevated concentrations of glucose in rat mesangial cells [53], we did not observe a significantly increase of USF2 in INS-1 832/13 cells grown under high glucose concentration suggesting the regulation of USF2 expression may be different between cell types. Despite the lack of increased USF2 mRNA level and USF2 protein accumulated in the nucleus in INS-1 832/13 cells maintained in the presence of high glucose, we observed the enhanced binding of USF2 to E4 in the ChIP assay, suggesting that posttranslational modification of this transcription factor may be responsible for the enhanced DNA binding activity. Nowak et al. [52] have reported that elevated concentrations of glucose stimulate expression of the apolipoprotein A5 gene through increased binding of USF1 and USF2 to their cognate E-box without changing protein abundance. Interestingly, this enhanced DNA-binding is at least in part caused by dephosphorylation of both USF1 and USF2. Therefore, it remains unclear whether USF2 and ChREBP co-regulate glucose-dependent transcriptional activation of the P2 promoter simply through competition of the same binding site.

It is also interesting to note that while all E-boxes function as glucose-responsive elements that mediate transcriptional activation of the P2 promoter under high glucose induction, E4 functions as a repressor under a normal glucose concentration as mutation of this E-box resulted in an increased reporter gene expression under a normal concentration of glucose. Jeong et al. [54] have recently reported that ChREBP can also function as a transcriptional repressor as well as an activator, however, a molecular explanation regarding this dual role of ChREBP was not investigated. Growing evidence has also indicated that in the promoters of glucose-responsive genes, a complex ChoRE may consist of the classical core ChoRE and the nearby accessory site which allows maximal glucose-induction. The accessory sites thus far have been reported to be the binding sites of the nuclear factor-Y (NF-Y) (i.e. CCAAT-box), HNF4α or c-myc [55], [56]. Jeong et al. [54] have recently employed integrated expression profiling and genome-wide analysis of ChREBP target genes and identified a guanine-rich sequence similar to a Sp1-binding site associated with ChREBP even though this sequence does not bind ChREBP. The authors suggest that this guanine-rich sequence may form part of an accessory site allowing Sp1-family transcription factors to bind and co-regulate with ChREBP. As mentioned above, we found that the CCCCCG sequence (i.e. GGGGGC on the complementary strand) coincided within the E2 and the ChREBP binding site (E4) and are in the close vicinity, which may be the case as reported by Jeong et al. [54].

Although, Sp1 has previously been known to be a ubiquitously expressed transcription factor which controls basal transcription of a variety of “housekeeping” genes [57]–[58], growing evidence now indicates that the abundance and its transcriptional activity of this transcription factor can be modulated by nutrients and cellular metabolites [59]–[63]. We have found that the CCCCCG sequence within E2 serves as a glucose-sensor because a high concentration of glucose increases the recruitment of Sp1 to this sequence. Also mutation of this sequence eliminated glucose-induced transcriptional induction of P2 promoter activity. We found that an elevated concentration of glucose did not affect the abundance of Sp1 mRNA or Sp1 protein in the nucleus but rather affects the phosphorylation status of the protein. We found that a high concentration of glucose increases dephosphorylation of threonine 453 of Sp1 and this might in part contribute to the enhanced transcriptional activity under this condition. Phosphorylation of this residue of Sp1 has been reported to decrease its ability to activate transcription of the cystathionine γ-lyase gene in pancreatic beta cells [63]. The regulation of PC expression by glucose appears to be similar to another biotin containing enzyme, namely acetyl-CoA carboxylase (ACC). In mouse preadipocytes, Sp1 also mediates glucose activation of the ACC1 gene via the two GC-rich sequences which form part of the glucose-responsive element [64]. Exposure of preadipocytes to high glucose stimulates dephosphorylation of Sp1 concomitant with an enhanced binding to its cognate binding site in the ACC1 gene promoter [41].

Finally the functional roles of USF1, USF2, ChREBP and Sp1 in transcriptional induction of endogenous PC expression were validated by an siRNA experiment. This clearly demonstrated that silencing expression of USF2, ChREBP and Sp1, but not USF1 mRNA, individually resulted in modest reductions of glucose-induced transcriptional induction of PC mRNA expression, suggesting that these three transcription factors may act in concert allowing maximal induction of PC in response to an elevated concentration of glucose.

The findings that glucose-induced transcriptional activation of PC in pancreatic β-cells is regulated by multiple transcription factors provide a complex paradigm in terms of the disease development because deregulation of some of these transcription factors in part underlies the impaired insulin secretion or hyperglycemia. For example, ChREBP was first known to link glycolysis and *de novo* fatty acid synthesis in liver. There is also a direct and strong association between ChREBP expression and insulin sensitivity in adipose tissue of humans with insulin resistance [65]. Growing evidence now indicates that this transcription factor also regulates transcription of β-cell specific genes [49]. Suppression of ChREBP expression impairs glucose-stimulated pancreatic β-cell proliferation [66]. Recently, glucose-induced ChREBP overexpression has been shown to play a pivotal role in mediating glucotoxicity of pancreatic β-cells, causing impaired insulin secretion [67]. Likewise deregulation of Sp1 via the abundance or its posttranslational modification is well known to at least in part cause diabetes [68].

In summary we identified a complex glucose-responsive unit which mediates glucose-induced transcription of the P2 promoter of the rat PC gene. Although this GRU appears to provide a platform for binding of at least four transcription factors, namely USF1, USF2, ChREBP and Sp1, only the latter three are functionally relevant to glucose-induced transcription of P2 promoter of rat PC gene.
